# Prediction of Refracturing Effect of Tight Gas Reservoirs Based on Bayesian Inversion Algorithm

**DOI:** 10.1155/2022/7593526

**Published:** 2022-05-11

**Authors:** Hai Lin, Fujian Zhou, Yakai Tian, Yan Wang

**Affiliations:** ^1^The Unconventional Oil and Gas Institute, China University of Petroleum (Beijing), Beijing 102249, China; ^2^PetroChina Qinghai Oilfield Company, Dunhuang, Gansu 736202, China

## Abstract

As a key technology for tight gas stimulation, refracturing plays an important role in tight gas development. In the production process of tight gas wells, the reservoir or fracturing process may cause the hydraulic fractures to gradually fail and the production to continuously decrease. In order to restore the productivity of a single well, it is necessary to refract the well to reopen the failed fractures or fracturing. Reasonable refracturing timing and optimization of refract fracture parameters are important guarantees to ensure the benefits of refracturing in tight gas wells, and relevant research on it can provide theoretical and technical guidance for field construction design. Based on the inverse problem of the dynamic prediction model of tight gas well productivity, this paper proposes an inversion method of formation and fracture parameters before refracturing based on Bayesian inversion algorithm. Finally, based on the geology and development data of the fractured wells in the Sulige gas field, the field application of refracting well selection, determination of refracting reasonable timing, and prediction of refracting effect is carried out. The actual production data are compared, and it is shown that this method can provide theoretical guidance for high-efficiency production-increasing construction on-site.

## 1. Introduction

In recent years, unconventional oil and gas has become a hot spot in oil and gas development at home and abroad. As an important part of unconventional gas reservoirs, tight gas reservoirs play an increasingly important role in oil and gas production enhancement [1]. The development of tight gas reservoirs is generally dominated by hydraulic fracturing. During the production process of gas wells, the reservoir or fracturing process may cause the hydraulic fracturing fractures to gradually fail and the production to decrease [2, 3]. In order to restore the productivity of a single well, refracturing is performed to reopen fractures that have failed or to open new ones. Refracturing, as a key technology to stimulate production in tight gas reservoirs, plays an important role in tight gas reservoirs [4]. Due to the long-term exploitation of tight gas and the continuous construction of gas wells in the gas field, the number of failed old wells has increased, and the physical properties of tight gas reservoirs have deteriorated and the stimulation effect has deteriorated [5, 6]. When refracturing gas wells in the gas field, refracturing too early will easily lead to the failure of the stimulation period of the previous fracturing to be fully utilized, which will affect the fracturing effect and the economic benefits of the gas field [7]. If refracturing is carried out too late, it is easy to cause the problem that the stimulation measures of the last fracturing cannot be replaced in time, resulting in loss of production increase. Determining the reasonable timing of refracturing is an important factor to ensure the effect of refracturing [8, 9]. In the process of gas field production, parameters such as well storage, skin, formation permeability, fracture half-length, and fracture conductivity cannot be accurately measured by instruments and equipment, which will affect the implementation of refracturing [10, 11]. Thus, it is necessary to invert these parameters, which provides a theoretical basis for the implementation of refracturing. In order to ensure that the production wells after refracturing have better productivity and economic benefits, it is necessary to predict the effect of refracturing. Optimizing the parameters of refracturing fractures can provide a theoretical basis for on-site construction design [12–14].

Foreign research on the selection of refracturing well layers is earlier, and after a lot of theoretical research and practice, a prediction method with considerable practical significance has gradually been formed. Some scholars have applied the typical curve method, statistical method, and artificial intelligence method to the selection of refracturing wells and layers and applied numerical simulation technology to compare the three methods [15, 16]. It was later concluded that the artificial intelligence method was the best forecasting method used at the time. At the same time, in the process of well selection and layer selection, they also conducted in-depth research on various methods to improve the accuracy of well selection and layer selection and then proposed that the combination of artificial intelligence and fuzzy logic algorithm can greatly improve the prediction accuracy [17–19]. Compared with the numerical simulation method, the results show that the accuracy of this method in the prediction of well selection and layer selection has been significantly improved. Some scholars believe that the effect of refracturing is the result of the comprehensive action of various influencing factors, and the neural network technology is very suitable for dealing with the problem of comprehensive impact evaluation. In addition, data mining technology was used for data analysis, and the regularity between the factors affecting the fracturing effect was found [20]. After using the data records of these main influencing factors to train and test the neural network model, they compared the predicted yield with the current yield, and the results showed that the neural network predictions were very close to the actual yield [21].

Compared with foreign research, domestic research on this technology started relatively late, and the focus of the research is mainly on the use of fuzzy mathematics and artificial neural network technology for classification and recognition. Although some researchers have studied the factors affecting the fracturing effect, they have not quantitatively analyzed these factors [22, 23]. Another part of the researchers realized the importance of quantitative analysis of influencing factors, and their research focused on the quantitative analysis of the main factors: perforation, construction methods, well entry materials, construction parameters, etc., so their research has more important significance. Some scholars in China have studied rapid well selection and formation evaluation methods suitable for mines and fields other than mines [24]. They have analyzed the production performance under different fracture lengths and have carried out rapid evaluation of reservoirs under this premise. The determination of the lower limit of the physical properties of the reservoir plays an important role in improving the effectiveness of the refracture in the refracturing of oil and gas wells. In addition, they also studied the fuzzy identification method of refracturing well selection and layer selection [25]. Using fuzzy identification method to optimize the fracturing well layer, the results show that their prediction method has a good effect. In this paper, based on the inverse problem of the dynamic prediction model of tight gas well productivity, a Bayesian inversion algorithm is proposed to invert the formation and fracture parameters before refracturing, and an empirical analysis of the Sulige tight gas reservoir is carried out.

## 2. Materials and Methods

### 2.1. Research Object

The Sulige gas field is located in the Suligemiao area in the city of Ordos. The lithologic gas reservoirs in the Sulige gas field are characterized by low permeability, low pressure, low abundance, and wide distribution. The sand body reservoirs have poor continuity, resulting in low single-well controlled reserves, low production, great difficulty in production, and poor stable production. Despite the application of gas recovery technology measures such as foam drainage and gas lift recovery, improving the development efficiency of gas fields still faces great challenges. It has a high proportion of low-yield and low-efficiency wells, and the pressure to increase and stabilize production is high. With the deepening of geological understanding and technological advancement, some old wells have the potential to increase production. Refracturing can achieve the purpose of increasing single-well production.

### 2.2. Research Method

#### 2.2.1. Determination of Reasonable Refracturing Time

Based on the selection of wells for refracturing, three methods are summarized to determine the reasonable timing of refracturing: well test data method, statistical law method, and dynamic analysis method.

The well test data method judges fracture failure or formation plugging according to the transient pressure testing curve before refracturing, so as to determine the reasonable time of refracturing. The characteristic of the transient pressure testing curve in dual-media formation is that the wellbore has a short storage time, and the double-logarithmic curve shows a double-track shape after the transition period. The characteristic of the well test curve of unstable homogeneous formation is that the gas wellbore has a long reservoir time, and the pressure derivative curve presents a horizontal straight line in the later stage.

The statistical law method uses statistics on the relationship between the time interval between refracturing and initial fracturing and the gas increase in gas wells.

The well test data method has uncertainties. For example, changing the working system of gas wells, perforation, etc. can lead to the same effect. The statistical law method is only for a specific block and does not have broad applicability. Therefore, this section adopts dynamic analytical method to determine the reasonable time for refracturing.

After the initial fracturing of the gas well, the existence of fracturing fractures changes the seepage mode of fluid in the formation. The production characteristics of fractured wells can be divided into fracture flow section, transition section, quasi-radial flow section, and boundary characteristic section.

1. Fracture characteristic section. At this stage, the natural gas flows to the wellbore through fractures, showing linear flow, and bilinear flow.
2. Transition section. The pressure diffusion range increases gradually, and the transition from fracture flow to quasi-radial flow.
3. Quasi-radial flow section. With the extension of production time, the influence of fractures is weakened and quasi-radial flow section appears.
4. Boundary reflection segment. When the time is long enough, boundary features appear.

For most well testing wells, the characteristics of linear flow and quasi-radial flow in the test curve are common, and the boundary characteristics are rare.

For tight sandstone gas, well test shall be conducted after initial fracturing. When the gas well production is lower than the industrial standard, conduct secondary well test after excluding non-fracturing production reduction factors. Compare the changes of fracture parameters of two well tests, so as to determine the best time of refracturing of the well.

#### 2.2.2. Genetic Method Data Inversion

Based on the inverse problem of the productivity prediction model, the parameter inversion method based on the genetic algorithm is used to solve the parameters such as skin, relative conductivity of fractures, half-length of fractures, and formation permeability in the stimulation area before refracturing. The specific steps are as follows:

(1) Set the fitness. The absolute value of the difference between the theoretical production capacity curve and the actual production capacity curve is used as the objective function. The fitness here is the optimization problem of the minimum value of the objective function; that is, the smaller the fitness, the closer the production capacity curve is to the actual output. The formation parameters and fracture parameters obtained by solving the inverse problem of the productivity model are closer to the actual values of tight gas reservoirs.
(2) Set the search range, group size, and parameter precision requirements. The calculation formula of binary code length *L* is as follows:
  (1) $$\begin{array}{c} 2^{L-1}<\frac{x_{\text{max}}-x_{\text{min}}}{\delta }+1<2^{L}. \end{array}$$
  (i) According to experience, the skin, fracture conductivity, fracture half-length, and formation permeability are, respectively, given a reasonable range.
(3) Generate a set of initial populations. Take a set of suitable initial values for skin, fracture conductivity, fracture half-length, and formation permeability.
(4) Copying with decimal parameters, this paper adopts the competitive merit-based method based on roulette selection.
(5) Given a specific exchange probability, randomly select the required exchange individuals. The binary encoding formula after the parameters are interpolated within the search range is
  (2) $$\begin{array}{c} x=x_{\min}+\frac{x_{\max}-x_{\min}}{2^{L}-1}\text{Dec}\left(y\right). \end{array}$$
  (ii) In the formula, *X*_min_ and *X*_max_ are the search range of each parameter; Dec is the conversion of binary to decimal; *X* is the actual value of the parameter; *L* is the binary number of *X*.
(6) Given a specific mutation probability Pm, randomly select the mutation position.
(7) If the fitness of the objective function is not achieved, repeat steps (3)-(6) until the desired fitness is reached.

#### 2.2.3. Establishment of Bayesian Algorithm and Its Inversion Method

*(1) Bayesian Method*. When the traditional parameter inversion method performs parameter inversion, although the optimal solution that satisfies the objective function can be obtained, the solution cannot be evaluated, and multiple optimal solutions may appear. Compared with deterministic inversion theory, Bayesian inversion quantitatively evaluates the uncertainty of inversion results and the relationship between model parameters by extracting the marginal probability distribution of model parameters, the maximum a posteriori solution, the average solution, and the correlation coefficient. The posterior probability distribution of model parameters reflects the constraining ability of observation data and prior information on model parameters. In this paper, the Bayesian inversion algorithm is extended to the reservoir parameter inversion. For the parameter inversion problem before refracturing, a parameter inversion method based on the Bayesian inversion algorithm is proposed in this paper. Well storage, skin, fracture conductivity, fracture half-length, and formation permeability before refracturing in tight gas wells can be derived.

The Bayesian parameter stochastic inversion method is a check calculation method that reduces the uncertainty of parameters and accurately estimates the delay probability density function of parameters by fusing multi-source information such as experimental data, observation information, and engineering experience which can be calculated as follows:

(3) $$\begin{array}{c} \sigma \left(m|d\right)=\frac{p\left(d|m\right)p\left(m\right)}{p\left(d\right)}. \end{array}$$

*M* is the model parameter and *d* is the observed data. *P* (*m*) is the prior probability distribution of model parameters; p(d) is the prior probability distribution of geological conditions which can be a constant. *P*(*d*/*m*) is the likelihood function. *σ*(*m*/*d*) is the posterior probability of the model parameter *m* to the observation data *d*.

The size of the likelihood function *P*(*d*/*m*) in the Bayesian inversion calculation reflects the degree of mismatch between the model response and the observed data. Usually, there is always some noise in the actual observation data, and the inversion model results will also have errors. When we assume that the distributions of these noises and errors belong to the Gaussian distribution, the likelihood function *P*(*d*/*m*) is equivalent to *r* the function exp[−SE(*m*)]; that is,

(4) $$\begin{array}{c} p\left(d|m\right)\propto \text{exp}\left[-S\cdot E\left(m\right)\right]. \end{array}$$

In the formula, *E*(*m*) is the objective function and *S* is the scale factor.

The objective function can be defined in various forms, and under the assumption that the data error is Gaussian distributed, the objective function we generally choose (called energy in simulated annealing) is expressed as

(5) $$\begin{array}{c} E\left(m\right)=\frac{1}{2}{\left[d-g\left(m\right)\right]}^{T}\times C^{-1}\left[d-g\left(m\right)\right]. \end{array}$$

In the formula, *g*(*m*) is the result obtained by the forward modeling of the model and *C* ^−1^ is the inverse of the data covariance matrix, including the observation error and simulation error of each model.

*(2) Establishment of Parameter Inversion Method Based on Bayesian Inversion Algorithm*. In this paper, the parameter inversion method based on Bayesian inversion algorithm is based on Bayesian theory and combined with simulated annealing algorithm. The most important thing in the inversion process is to have a large number of sampling model samples, which is convenient for posterior probability calculation as well as obtaining the mean and variance of the model by statistical samples.

The solution of the production capacity prediction model is a positive problem. Here, the inverse problem of the production capacity of the five zones is solved, and the parameter inversion method based on the Bayesian inversion algorithm is used to solve the skin, the relative conductivity of fractures, the half-length of fractures, and the permeability of the reformed area. On the whole, Bayesian inversion can be divided into three parts: the collection and transformation of prior information, the inversion calculation and the collection of sample models, and the statistics of model parameters. The specific inversion process is as follows:

1. Collect the prior information of parameters such as skin, relative conductivity of fractures, half-length of fractures, and permeability of reformed area, and transform them into prior probability density distribution and constraint conditions after analysis and processing
2. Randomly generate model parameters under the constraints of these four parameters
3. Use the production capacity prediction model to perform forward modeling, calculate the objective function of the initial model, and save the model sample and its posterior probability value if the model satisfies the Metropolis criterion in the simulated annealing algorithm
4. Repeat steps (2) and (3) to sample a large number of samples
5. Judging the termination conditions, if the termination conditions are met, output all model samples and their corresponding posterior probabilities
6. Uniform the posterior probability distribution of all model samples obtained by inversion to ensure that the sum of the posterior probability values of all samples is 1
7. Calculate the mean and variance of the skin, relative conductivity of fractures, half-length of fractures, and permeability of the reformed area
8. Output the mean, variance, and posterior probability distribution of these four parameters

## 3. Results and Discussions

### 3.1. Method Comparison

A conceptual model of a fracturing vertical well was established by numerical simulation software, and the fracturing fractures were simulated by the local grid refinement method. According to the average control range of single well in Sulige gas field, the length and width of the reservoir were both 700 m and 20 m thick, the original formation pressure was 15 MPa, the porosity was 10%, and the formation permeability was 0.0001 mD. The half-length of the main fracture is 135 m, the width of the main fracture is 0.008 m, the permeability of the main fracture is 10 mD, and the skin coefficient is 15, and the grid step size is d*x* = d*y* = 15 m, d*z* = 10 m. The well was produced at a fixed bottom hole pressure of 5 MPa.

#### 3.1.1. Genetic Method Data Inversion

**Figure 1:**
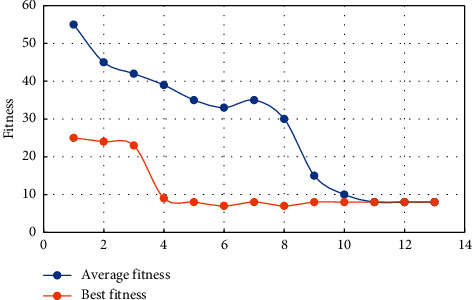
The graph of fitness changing with genetic algebra.

We set the population size to 30, exchange probability to be 0.9, and mutation probability to be 0.002. The maximum number of iterations in each cycle is 13, and the inversion results are shown in Figure 1, where the abscissa genetic algebra is equivalent to the number of iterations, and the ordinate fitness is equivalent to the objective function. It can be seen from Figure 1 that the fitness changes with the genetic algebra, the optimal fitness value obtained by the parameter inversion algorithm based on the genetic algorithm, that is, the optimal target value, is 7.02, the average fitness is 8.40. The parameters of the current optimal individual values are 0.25 for the skin, 40.00 m for the half-length of the fracture, 15.64 mD for the permeability of the reformed area, and 38.7 for the relative conductivity of the fracture.

#### 3.1.2. Parameter Inversion Based on Bayesian Inversion Algorithm

**Figure 2:**
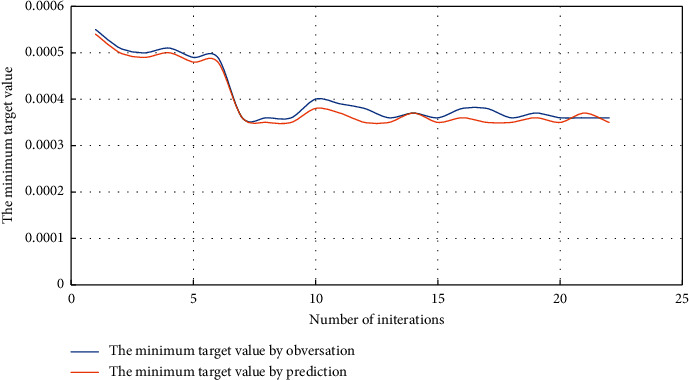
The curve of the minimum target value with the number of iterations.

The parameter inversion method based on the Bayesian inversion algorithm proposed in this paper is used to invert the skin, fracture relative conductivity, fracture half-length, and permeability of the stimulation zone before refracturing of fracturing vertical wells and determine the range of initial model parameters. The skin is between 0.1 and 30, the half-length of the fracture is between 60 and 200 m, and the fracture permeability in the reformed area ranges from 0.2 to 1000 mD. The relative conductivity of the fracture is ranging from 1 to 70, and the number of iterations is set to 50. The objective function is the sum of the absolute value of the production difference at each point on the production capacity prediction curve and the actual production capacity curve. The minimum target value changes with the iteration times as shown in Figure 2.

In order to compare the parameter inversion algorithm based on the genetic algorithm and the parameter inversion method based on the Bayesian inversion algorithm, this paper compares the two parameter inversion methods from the size of the target value and the degree of closeness to the actual value of the parameter (Table 1).

### 3.2. Case Study

**Figure 3:**
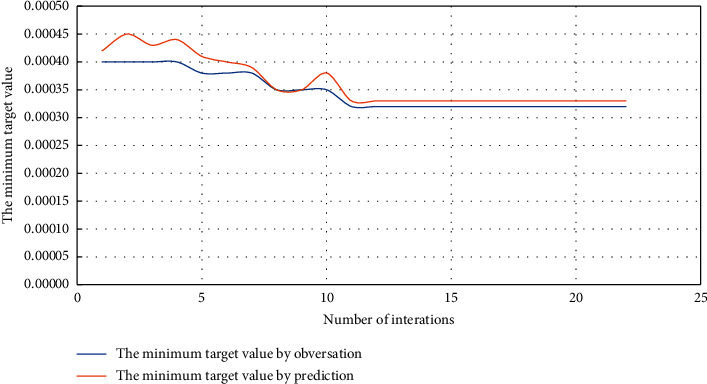
Curve of minimum target value with iteration times.

A block in the Sulige gas field is a tight reservoir with poor overall physical properties and complex pore structure, with a porosity of 5%–12% and a permeability of 0.03–1.2 mD. It has the characteristics of low porosity and ultra-low permeability reservoir. In this paper, one of the production wells, Well *M*, is taken as the research object. Based on the productivity prediction model, the dynamic analysis method is used to determine the reasonable timing of its refracturing. Collecting and analyzing the geological development data of this well, the reasonable time for the refracturing of Well *M* is 2000 days; that is, the refracturing is carried out 5 and a half years after production. The parameter inversion method based on Bayesian inversion algorithm is used to solve the skin, relative conductivity of fractures, half-length of fractures, and permeability of the stimulation zone before refracturing of the well. The inversion calculation diagram is shown in Figure 3. Figure 3 shows that the skin is 7.59, the half-length of the fracture is 40.21 m, and the relative conductivity of the fracture is 28.95.

**Figure 4:**
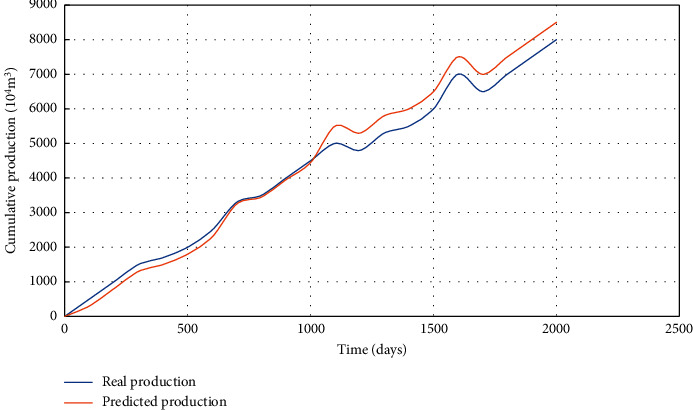
Predicted curve and actual curve of cumulative gas production by refracturing in Well M.

Based on the inversion results of refracturing parameters in Well *M*, the prediction of refracturing effect in Well *M* is carried out. According to the data of formation fluid properties, high-pressure physical property parameters, and phase permeability curve, numerical simulation of refracturing is carried out for Well *M*. By refracturing well *M*, the effect of refracturing is predicted at this time, and the predicted and actual curves of cumulative gas production are shown in Figure 4. The cumulative gas production prediction curve and the actual curve have a high degree of fitting, indicating that the prediction of refracting effect can provide theoretical guidance for the design of refracting in gas fields and improve economic benefits.

## 4. Conclusion

1. In this paper, a Bayesian inversion algorithm-based inversion method for formation and fracture parameters before refracturing is proposed, and the inversion results are compared with the inversion algorithm based on genetic algorithm. The parameter inversion method of the Bayesian inversion algorithm has great advantages and high prediction accuracy.
2. It is proved by field practice that the predicted curve of cumulative gas production has a high degree of fitting with the actual curve, indicating that the prediction of refracting effect can provide theoretical guidance for the design of refracting construction in gas fields, improve economic benefits, and further explain that the Bayesian inversion algorithm can predict the effect of refracturing in tight gas reservoirs.

## Figures and Tables

**Table 1:** Comparison of parameter inversion results based on genetic algorithm and Bayesian inversion algorithm.

Method	Skin	Fracture half-length (m)	Fracture relative conductivity	Target (m^3^)
Actual parameter value	15	135	39	—
Genetic algorithm	0.25	40	38.7	36
Bayesian inversion algorithm	16.75	151.5	30	0.0004

## Data Availability

The figures and tables used to support the findings of this study are included in the article.
